# Making Blood from the Vessel: Extrinsic and Environmental Cues Guiding the Endothelial-to-Hematopoietic Transition

**DOI:** 10.3390/life11101027

**Published:** 2021-09-29

**Authors:** Wade W. Sugden, Trista E. North

**Affiliations:** 1 Stem Cell Program, Department of Hematology/Oncology, Boston Children’s Hospital, Boston, MA 02115, USA; wade.sugden@childrens.harvard.edu; 2 Developmental and Regenerative Biology Program, Harvard Medical School, Boston, MA 02115, USA

**Keywords:** hemogenic endothelium, HSCs, EHT, zebrafish, blood flow, hematopoiesis

## Abstract

It is increasingly recognized that specialized subsets of endothelial cells carry out unique functions in specific organs and regions of the vascular tree. Perhaps the most striking example of this specialization is the ability to contribute to the generation of the blood system, in which a distinct population of “hemogenic” endothelial cells in the embryo transforms irreversibly into hematopoietic stem and progenitor cells that produce circulating erythroid, myeloid and lymphoid cells for the lifetime of an animal. This review will focus on recent advances made in the zebrafish model organism uncovering the extrinsic and environmental factors that facilitate hemogenic commitment and the process of endothelial-to-hematopoietic transition that produces blood stem cells. We highlight in particular biomechanical influences of hemodynamic forces and the extracellular matrix, metabolic and sterile inflammatory cues present during this developmental stage, and outline new avenues opened by transcriptomic-based approaches to decipher cell–cell communication mechanisms as examples of key signals in the embryonic niche that regulate hematopoiesis.

## 1. Introduction

Endothelial cells of the vascular tree represent the primary interface between the organs of the body and the blood, which supplies tissues with nutrients, oxygen and other compounds from the environment to maintain homeostasis. The cellular constituents of the blood, oxygen-carrying erythrocytes and the lympho-myeloid cells of the immune system, are generated and replenished throughout life by a population of multipotent hematopoietic stem and progenitor cells (HSPCs). The relationship between blood and vessel is intimate indeed: during embryonic development of vertebrates, definitive HSPCs are derived from hemogenic endothelium (HE) of select large arteries via a morphological and transcriptional transformation termed the endothelial-to-hematopoietic transition (EHT). Given the clinical value of hematopoietic stem cells (HSCs), which can reconstitute the full blood system upon transplant in a number of human disease states, much effort has been devoted to understanding the cellular and molecular mechanisms governing EHT to allow development of rational protocols to expand patient HSC numbers or derive them de novo from induced pluripotent stem cell (iPSC) sources. The zebrafish model has been a cornerstone of this effort, owing to the high degree of conservation with mammalian systems and the ability to perform large-scale genetic and chemical screens that have revealed both intrinsic and extrinsic factors necessary for vertebrate hematopoiesis.

## 2. The Endothelial Origin of HSCs

### 2.1. Definitive Hematopoiesis in the Embryo

**Figure 1:**
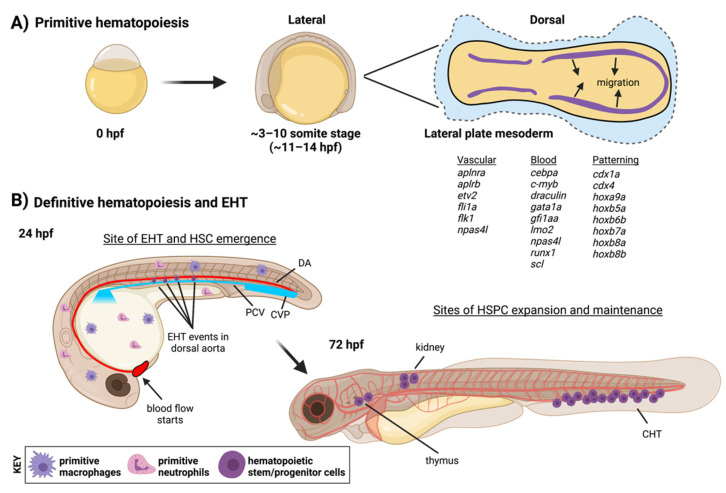
Stages and anatomical locations of hematopoietic development in zebrafish. (**A**) In the first 24 h of development, mesoderm is specified from which all subsequent blood and endothelial cells will emerge (see gene lists for markers of different mesodermal populations). The primitive wave of hematopoiesis generates early red blood cell and immune populations. (**B**) Over the next two days in a definitive wave of hematopoiesis, HSCs will be produced from HE in the dorsal aorta by EHT and seed distant hematopoietic niches for expansion and lifelong blood production. EHT endothelial-to-hematopoietic transition, CHT caudal hematopoietic tissue, CVP caudal vein plexus, DA dorsal aorta, PCV posterior cardinal vein. Figure created with BioRender.com.

Endothelial and blood cells share a common ontogeny during development, arising from the mesodermal germ layer after gastrulation. Here, we will emphasize zebrafish-specific developmental stages, timing and markers to highlight areas of high conservation with other vertebrates as well as aspects peculiar to the zebrafish model to underscore the current state of our understanding of this process. All blood and vascular lineages are derived from cells in the lateral plate mesoderm (LPM). These structures are present as stripes in the zebrafish embryo (Figure 1) from ~11–14 h post fertilization (hpf) [1]. Positional information helps to specify different mesodermal derivatives along the A-P axis, but in zebrafish the *cloche/npasl4* transcription factor (TF) has been shown to be essential for the proper development of both blood and endothelial cells from this tissue [2]. LPM cells migrate to the midline where vascular progenitors will segregate and coalesce to form the two primary axial vessels: the dorsal aorta (DA) and posterior cardinal vein (PCV). Restricted blood progenitors from the LPM produce primitive erythrocytes to fill these new vessels, while the embryo is patrolled by the first innate immune cells, macrophages and neutrophils. These first blood cells descend from committed progenitors that are restricted to either the erythroid or myeloid lineages, and this event is referred to as the primitive wave of hematopoiesis [3]. In contrast, from ~24–48 hpf HSPCs will be produced exclusively from arterial endothelium by an EHT or hematopoietic “budding” event. In mammals, EHT-dependent HSPC production occurs primarily along the DA within the aorta-gonad-mesonephros (AGM) region, analogous to zebrafish, with contributions from additional embryonic and extraembryonic arteries [4]; blood production at this stage is referred to as the definitive wave of hematopoiesis. HE cells are restricted to the ventral wall of the DA, and expression of the same hematopoietic TFs that orchestrate the transcriptional program driving EHT in mice, including Gata2 [5], Runx1 [6], and cMyb [7], can be used to identify HE in zebrafish. The capacity of cells formed via EHT to renew themselves or generate cells of the erythroid, myeloid and lymphoid lineages throughout the lifetime of the animal distinguishes select progenitors as HSCs. It is important to note that more lineage-restricted definitive progenitors have been documented to arise via EHT events both before and concurrent with HSC emergence, and new transcriptomic and lineage-tracing data from zebrafish suggests that embryonic blood production primarily occurs from these cell types (with the HSC pool being drawn on later in adult life) [8]. These include lymphoid progenitors from the caudal DA in zebrafish [9,10], murine lympho-myeloid progenitors [11,12] and erythro-myeloid restricted progenitors (EMPs) in the zebrafish trunk [13] and from yolk sac arteries in mice [14]. A recent report even suggests that yolk-sac derived EMPs may produce endothelial cells that can incorporate back into the developing embryonic vasculature [15]. Thus, taken together it is clear that the development of the hemato-vascular system involves plastic and not necessarily unidirectional fate decisions. Over the next few days of development, HSCs produced during the definitive wave will colonize embryonic niches to expand and reside for lifelong blood production: these sites include the caudal hematopoietic tissue (CHT), the thymus and kidney marrow [16]. As assays currently available in zebrafish to determine the multi-lineage and self-renewal potential of a given blood stem cell are somewhat limited, the more conservative term HSPCs is often applied to the populations residing in these embryonic niches.

As in many fields of study, unique advantages of the zebrafish model have propelled the investigation of hematopoiesis. The external fertilization and optical clarity of zebrafish embryos in particular allowed the first ever capture of the dynamic components of blood development. Live-imaging studies elegantly showed the migration of LPM cells to the midline to form blood and vessels [17,18,19], previously inferred from static images of gene expression [20]. Further work has shown that cues are provided from the somitic tissue and other nearby cell types during this migration that impact on later HSPC production [21,22,23]. Live-imaging in zebrafish was also able to unequivocally demonstrate the generation of HSPCs from aortic endothelium via EHT: time-lapses document the physical and fate changes of Flk1^+^ aortic endothelial cells as they round up, express hematopoietic transgenes, detach from the adjacent endothelium and transmigrate through the neighboring PCV to circulate through the embryo [24,25]. This process is well conserved in mammals, and can be imaged in situ following terminal embryonic dissection in mice [26]. Aside from live-imaging, the fruitfulness of large-scale genetic screens for isolating anemic mutants and substantial genetic and cellular conservation between zebrafish and mammals has identified many of the molecular players involved in all stages of blood development [27,28]. Importantly, many of the growth factors that are responsible for vascular development and patterning also play roles in HSPC production in zebrafish, including VEGF [29], BMP [30,31], FGF [32,33], WNT [34] and TGFβ [35]. Likewise, secreted factors such as cKit and OncostatinM, which are linked specifically to hematopoietic development in mammals, were recently shown to exhibit a pro-hematopoietic function via knockdown studies in zebrafish [36].

### 2.2. Importance, and Limits, of Intrinsic Factors

The importance of cell intrinsic regulators, particularly TFs, in generating HSCs from HE cannot be overstated. Indeed, the master regulator Runx1 is required for EHT: mice [6,37] and zebrafish [38] deficient for Runx1 fail to undergo budding and produce definitive HSCs. Several key hematopoietic TFs work in concert to orchestrate the transcriptional rewiring from endothelial to hematopoietic identity [39]. In zebrafish, recent studies have clarified roles for *gata2a* [40], *gata2b* [41], *gfi1aa* and *gfi1b* [42], and Notch receptors [43,44] in specifying a competent population of HE in the embryonic DA. A summary of the proposed transcriptional hierarchy in zebrafish HE from these studies is as follows: a Notch-responsive population of *gata2a*-expressing arterial endothelium in the ventral wall of the DA acquires the expression of *gata2b* (in a *gata2a*-dependent fashion). This TF in turn promotes *runx1* expression, which itself induces *cmyb* expression. *Runx1* and *cmyb* together ensure a downregulation of the arterial transcriptional program, an enduring commitment to hematopoietic fate and the successful completion of EHT. This hierarchy, and ultimate ‘arterial-to-hematopoietic’ transcriptional shift, is broadly conserved in murine hematopoiesis [45]. Notably, the distinct biology of embryonic zebrafish can reveal molecular potential difficult to study in mouse embryos due to embryonic lethality and in utero development. For example, although Runx1 loss of function is lethal in mice, up to 20% of homozygous *runx1 -/-* zebrafish can make it to adulthood under the right laboratory conditions [46]. Due to their small size, passive diffusion of O_2_ from aqueous growth medium can allow zebrafish embryos to survive cardiovascular and hematopoietic insult that would kill mammalian embryos [47]. Recent data suggest that, in the absence of *runx1*, the *gata2a* and *gata2b* genes in zebrafish can coordinate a form of ‘salvage hematopoiesis’ to produce enough blood progenitors to enable the survival of *runx1 -/-* animals [48]. The authors show that mouse adult HSPCs also upregulate Gata2 when Runx1 is lost, suggesting the transcriptional logic may be conserved. In parallel to studies of hematopoietic TFs, morpholino and mutagenesis screens in zebrafish have investigated the functions of epigenetic regulators and chromatin factors in creating a permissive nuclear environment for hematopoietic TFs to successfully direct EHT. The DNA methyltransferases *dnmt1* [49], *dnmt3bb.1* [50], histone deacetylases *hdac1* [51], *hdac6* and *hdac9a/b* [52], chromatin remodeler *smarca5* [53] and members of the polycomb repressive complexes 1 [54] and 2 [55,56] all have zebrafish data supporting their roles in the regulation of EHT genes.

Discovery of the endothelial origin of definitive blood stem cells in vertebrates has raised key questions regarding the necessity and sufficiency of the endothelial state passed by mesodermal cells destined for hematopoietic specification. Findings from in vitro reprogramming provide provocative indications that hematopoietic progenitors can be generated from non-endothelial sources [57,58], or without passing through arterial intermediate states [59,60]. However, strategies that bypass normal developmental ontogeny of HSCs produce cells that fail to fully recapitulate the transcriptional signatures of their in vivo counterparts [61], and simple overexpression of TF cocktails yields modest numbers of hematopoietic cells from iPSCs, which have limited engraftment and reconstitution potential [62,63]. Recent data support that a Dll4^+^, Notch-responsive endothelial population is most effective at producing multipotent blood cells in culture [64,65], and these data together highlight that intrinsic factors alone cannot fully recapitulate the complex cell–cell and cell–environment interactions that occur to generate HSCs during EHT. The zebrafish model has been successfully leveraged to fill this gap in knowledge and identify several extrinsic cues in the embryonic environment that might improve efforts to generate and expand HSCs. Admittedly, a notable deficit in the investigational toolbox for zebrafish researchers is that of standardized reprogramming or differentiation protocols that yield zebrafish hematopoietic cell types under defined conditions in vitro. One technical reason for this is the species-specific divergence in structure and function of crucial cytokines, which is known to underlie differential requirements for particular ligands and receptors between murine and human immunological systems [66]. It is possible to successfully culture zebrafish blood progenitors (either adult or embryonic) on preparations of zebrafish kidney marrow stromal cells to provide a supportive signaling milieu [67], or apply colony-based assays to quantify clonal progenitor potential using recombinant zebrafish cytokines in growth media supplemented with a fish serum [68,69]. However, currently, there is not broad commercial support for these resources, inhibiting their widespread dissemination and application. Limitations notwithstanding, attempts to import factors deemed to govern zebrafish HSC development in vivo to the more established non-fish culture systems as a means of determining conservation and function have proven successful. One such effort yielding promising biomedical translation was a chemical-screening approach that identified prostaglandin E2 as a positive regulator of HSPC production in zebrafish embryos [70], leading to subsequent clinical trials and therapeutic use for human HSC transplantation [71]. We review here some of the many other extrinsic signals guiding HSC development that have emerged in the last 10 years.

## 3. Extrinsic Cues: Mechanical Forces in the Environment

### 3.1. Influence of the Extracellular Matrix in EHT

The biophysical properties of a cell’s microenvironment or substrate are known to impact differentiation capacity and cell fate. These properties are a function of the composition of the extracellular matrix (ECM) in which the cell is embedded or adjacent [72]. Likewise, the intrinsic physical properties of a (stem) cell and its ability to exert forces against the ECM and/or other external stimuli can play a role in homeostatic and biological functions [73]. For example, HSCs of global mouse protein tyrosine phosphatase *Ptpn21* mutants have decreased cell stiffness and are more easily deformed, manifesting in hematopoietic phenotypes of enhanced egress from the bone marrow and failure to reconstitute the blood upon transplantation [74]. These biomechanical and physiological defects can be restored upon overexpression of a non-phosphorylatable variant of the Ptpn21 target, Septin 1, highlighting that physical stimuli are relevant cues throughout the life of an HSC.

**Figure 2:**
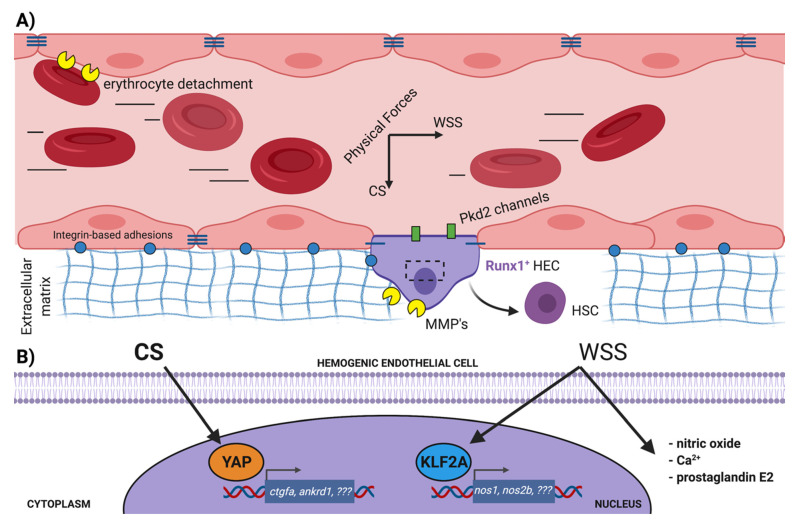
Mechanical cues in the aortic microenvironment impacting HSC production via EHT. (**A**) With the onset of blood circulation in the dorsal aorta, Runx1-expressing hemogenic endothelial cells are subject to the perpendicular hemodynamic forces of wall shear stress and cyclic stretch. Additional cues are presented by the extracellular matrix, which must be remodeled to allow the extravasation of newly minted HSCs and the completion of EHT. Other mechanical stimuli may be mediated by the content of the plasma itself, i.e., blood viscosity and cellular composition, but this requires further investigation. (**B**) WSS and CS have unique intracellular effects in HE. WSS upregulates expression of the TF KLF2A, which promotes expression of nitric oxide synthases. WSS also stimulates production of prostaglandin E2 and calcium influx, both with pro-hematopoietic effects on EHT. CS directly induces nuclear localization of the YAP TF, stimulating expression of its canonical target genes via mechanotransduction. Other genes that might be regulated by these TFs to orchestrate EHT remain to be identified. CS cyclic stretch, EHT endothelial-to-hematopoietic-transition, HEC hemogenic endothelial cell, HSC hematopoietic stem cell, MMP matrix metalloproteinase, TF transcription factor, WSS wall shear stress. Figure created with BioRender.com.

During the EHT process, the nascent HSCs produced from the DA delaminate from their neighbors, assemble into clusters of hematopoietic cells and ultimately break intercellular adhesive bonds to enter circulation as single cells, Figure 2. This extrusion requires detachment from, and remodeling of, the ECM as well. Using zebrafish, Theodore and colleagues [75] showed functions for the matrix metalloproteinases Mmp2 and Mmp9 in regulating developmental hematopoiesis. They found that knockdown of *mmp2* led to accumulation of cells in hematopoietic aortic clusters, due to an overabundance of fibronectin-rich ECM. Subsequently, Mmp9 controls retention of HSPCs in the CHT niche by degrading the chemokine Cxcl12. Another metalloproteinase, Adam8, functions at even earlier stages, promoting the detachment of the first primitive erythrocytes from the vessel wall in zebrafish to establish blood circulation [76].

Membrane-bound integrins are key molecules interfacing between both cell–cell contacts and cell–ECM adhesion, and mediate physical effects of both inputs on HE cells. They can participate in adhesion with hundreds of ligands, including other integrins and surface proteins on neighboring cells to form epithelial sheets and facilitate immune cell capture and extravasation, as well as many of the secreted proteins that make up the ECM [77]. A role for *itgb1b* in proper HE specification has been demonstrated in zebrafish. As previously mentioned, during the migration of LPM cells toward the midline signaling from the somitic tissue instructs some degree of hematopoietic competence in select endothelial cells before they coalesce and form the axial vessels. The strength of this signal is thought to be controlled, in part, by the strength of the adhesion of the Flk1^+^ mesoderm to the somite [22]. In a meticulous characterization of zebrafish mutants for the Rap1b GTPase, Rho et al. [78] showed defects in Runx1^+^ HE, as well as reduction in levels of the early HE marker *gata2b*. They could show that these effects were phenocopied in zebrafish *itgb1b* mutants as well and propose a mechanism whereby Rap1b activity induces Itgb1b-dependent adhesion to fibronectin to mediate close association of LPM cells with the somite and ensure a sufficiently strong induction of Notch signaling to specify HE. More recently, Li et al. used live-imaging and genetic analysis to show a requirement for *itga4* in HSPCs to allow their arrest and retention in the caudal hematopoietic tissue (CHT) in the embryo at 72 hpf [79], via interaction with VCAM1^+^ macrophages. Further study will likely clarify which integrins are required during the EHT process itself, and through what ECM components these effects are dependent.

### 3.2. Role of Blood Flow in Promoting EHT

The two landmark studies that first established the importance of hemodynamic forces as a cue that regulates hematopoiesis from endothelial cells in mice and zebrafish both identified nitric oxide (NO) as a potent small molecule induced by shear stress that promotes HSC development [80,81]. A battery of publications from these labs, and others, further described prostaglandin E2 [82], PKA activation [83] and adenosine receptor expression [84] as cellular responses downstream of blood flow that have positive effects on HSC production. Excellent quantitative measurements of blood flow parameters in the zebrafish DA throughout early developmental stages have been generated in recent years [85,86,87]. At the relatively low-intensity shear stress levels found in the arterial tree during the window of EHT, primary cilia of endothelial cells participate in force-sensing, and the importance of this organelle to definitive hematopoiesis has been comprehensively shown in zebrafish using chemical, knockdown and knockout strategies of cilia ablation [88]. Nevertheless, understanding of the full range of force sensors in HE that respond to the physical forces of blood flow, or how this information is transduced into gene expression changes to promote EHT, is far from complete.

In 2011, Wang et al. [89] used morpholino experiments in zebrafish to position the transcriptional regulator Klf2a downstream of NO in regulating *runx1* expression in the DA. Full genetic knockout zebrafish for *klf2a* are viable and do not recapitulate these hematopoietic defects [90], though a comprehensive analysis of simultaneous knockout of *klf2a* and the zebrafish orthologue *klf2b* would be required to address possible compensation [91]. A zinc-finger TF, Klf2 is one of the most highly upregulated genes in endothelial cells exposed to laminar shear stress [92], and its loss leads to embryonic lethality in mice which present with hemorrhaging, vascular defects and high-output heart failure. Curiously, a study revisiting the lethality of *Klf2*-/- mice revealed that the proximal cause was likely defective heart valve formation. Goddard et al. [93] concluded that flow-induced Klf2 expression in the endocardium of the heart valves at E9.5 in mice led to the expression of Wnt9 which influenced non-cell autonomous proliferation and morphogenesis of the underlying mesenchymal cells in the cardiac cushion. In this study, control of *wnt9b* expression by Klf2a was conserved during zebrafish heart development as well, where Klf2a is also known to regulate fibronectin synthesis [94]. Notably, the WNT ligand Wnt9a was recently shown to be essential for the “amplification” of hemogenic endothelial cells in the zebrafish DA, via binding to the Fzd9b receptor [95,96]. While these studies clearly demonstrated that Wnt9b did not influence HE expansion and that the presumptive source of ligand were the somites, it is striking that the window of “amplification” is precisely from the beginning of blood flow onwards. Future studies may indeed identify Klf2-regulated growth factor expression from endothelial cells exposed to blood flow that act in an autocrine fashion to control hematopoiesis from the endothelium.

The YAP and TAZ TFs of the Hippo pathway are also well-positioned to transduce biomechanical signals from blood flow, given their known nuclear translocation following mechanical stimulus [97]. Although sustained laminar flow in adult vessels suppresses YAP nuclear localization [98], elegant chemical/genetic manipulation of the Hippo pathway and blood flow in zebrafish shows YAP nuclear localization in endothelial cells of the DA receiving the kinds of flow magnitude present during the EHT window [99]. In adult mice, genetic approaches have shown that neither overexpression of constitutively active YAP (driven by Mx1:Cre induction following polyI:C administration) [100] or combined deletion of YAP/TAZ in HSCs transplanted into otherwise WT irradiated hosts [101] appear to affect the number or function of cells in the stem cell compartment. Conversely, YAP is essential for the production of definitive HSCs via EHT during development. Goode et al. [102] employed a range of ‘omics approaches to curate an atlas of transcriptional and regulatory signatures across several developmental stages of murine embryonic stem cells cultured in vitro through hematopoietic differentiation toward a macrophage fate. Stage-specific analysis of enriched motifs at promoters indicated an increased signature of the DNA-binding protein TEAD (a canonical YAP cofactor) during the endothelial stages of hematopoietic differentiation. Functionally, blocking YAP/TEAD interactions with verteporfin inhibited the production of hematopoietic progenitors (HPs) from embryoid bodies in vitro or AGM-derived Flk1^+^ endothelium cultured ex vivo. Importantly, treatment of HPs themselves with verteporfin did not affect their numbers or survival, similar to observations in adult mice and indicating a requirement for YAP/TEAD in the endothelium prior to EHT. Using a novel ‘organ-on-a-chip’ platform for culture of human CD34^+^ endothelial cells and pharmacologic/genetic manipulation in zebrafish embryos, our group recently showed that cyclic stretching of the endothelium from embryonic blood flow activates YAP via Rho GTPases, leading to the expression of Runx1 and the production of HSPCs [103]. Critically, our in vivo experiments demonstrate that YAP is not absolutely required for the specification of hemogenic endothelium in the aorta or initiation of Runx1 expression, but that in the absence of YAP the hematopoietic program cannot be maintained. These effects are conserved across species and suggest that mimicking physical forces by chemical stimulation of YAP mechanotransduction may improve the generation of hematopoietic cells in culture. In support of this notion, a similar enhancement of HSPC numbers in the mouse DA and function in CFU assays was observed upon Mll1 overexpression, which was attributed to an increased transcriptional signature of Rho/Rac signaling [104].

### 3.3. Cell Contractility and Aortic Architecture during EHT

A number of outstanding but interesting questions remain to be resolved concerning cell intrinsic mechanics, the effects of external blood flow forces on EHT and the overall preservation of aortic patency whilst maintaining production of HSCs. Zebrafish treated with the ROCK inhibitor Y-27632, which decreases cellular contractility, were shown to have increased cMyb:EGFP^+^ HSPCs in the DA [105]. More recently, a detailed live-imaging study from Lancino and colleagues [106] concluded that actomyosin contractility is essential for successful EHT; they describe the formation of an anisotropic circumferential actomyosin belt and local enrichments of tight junction protein ZO-1 as extruding HE cells bring together non-hemogenic endothelial cells and facilitate new tri-junctional contacts before complete separation. In their study, knockdown of the myosin light chain proteins *myl9a* and *myl9b* reduced CD41:EGFP^+^ HSPC numbers in the embryo. These discrepancies may relate to the different transgenes used for quantification, which identify slightly different stages of hematopoietic commitment. Alternatively, they could be explained by the chemical vs. genetic approaches, and interpretations of fate vs. migration phenotypes. Curiously, manipulation of blood flow in the Lancino study led to the conclusion that the ‘no flow’ condition in silent heart morphants caused fewer EHT events to occur, and to actually reverse in direction in some cases (into the luminal space of the aorta instead of toward the vein). Seemingly contradictory results were obtained by Camphino et al. [107] while studying the cellular architecture and behavior of aortic endothelial cells in zebrafish. They found that extrusion events were slightly increased in embryos with no flow, but that overall physical movement and rearrangements of cells from the dorsal to ventral aorta were impaired. They identified the stretch channel Pkd2 as essential for the flow-dependent kinetics of extrusion, and propose that controlling the speed of this process might increase the time HE cells spend exposed to hemodynamic forces while they undergo a transcriptional fate change. Excitingly, empirical data from live imaging in zebrafish are being combined with mathematical modeling to predict tissue mechanics in the DA during HSPC formation which may instruct where these events occur [108,109]. Additionally, cell tracing studies, such as those recently described by Ulloa et al. [8], can help distinguish whether the different types of stem and progenitor populations that are produced by EHT may be differentially impacted by blood flow and/or intra- and extracellular structural dynamics. Future work may also clarify exactly when the flow-dependence of the EHT process is relieved, and precisely when the contractile machinery is used for morphogenetic movements vs. fate acquisition.

## 4. Extrinsic Cues: Metabolic, Hormonal and Inflammatory Signals

### 4.1. Glucose and Other Metabolites

Endothelial cells regulate diverse intracellular metabolic pathways to carry out their specialized functions during angiogenesis, vessel remodeling/maturation and quiescence/homeostasis of the adult vasculature [110]. It is notable that endothelial cells rely on glycolysis for the bulk of their energy production, despite direct contact with the O2-carrying blood. In addition to these observations in non-hemogenic endothelium, it has become clear that glucose metabolism has strong effects on the production of HSCs from HE. In 2013, Harris et al. [111] demonstrated that exposure to elevated glucose levels during development caused significant increases in HSPC production in zebrafish embryos, together with increases in cell cycling and acceleration of the acquisition of hematopoietic gene expression in the DA. These pro-hematopoietic effects were shown to be dependent on both glycolysis and aerobic respiration, and chemical/morpholino-inhibition experiments indicated that stimulation of the Hif1a TF by reactive oxygen species (ROS) generated during glucose metabolism is a driver of HSPC production in this setting. These findings, with respect to Hif1a, were recently replicated in TALEN/CRISPR-generated zebrafish mutants: homozygous *hif-1aa/ab* double mutants show reduced hematopoietic gene expression, and the authors could show additionally that *hif-2aa/ab* also contribute to EHT [112]. Endothelial-specific deletion of *Hif1a* in mice compromises HSC production in the AGM, demonstrating conservation across species [113]. Free glucose is not the only metabolic avenue by which HSPC production is regulated. Secretion of ApoA-I binding protein 2 from the somites promotes EHT in HE cells by increasing the activity and expression of the cholesterol synthesis master regulating TF, *srebf2* [114]. Mutants unable to deposit the m6A RNA modification [115] or with ribosome biogenesis deficiencies [116] have reduced HSPC output.

Last year, the Nicoli Laboratory published a comprehensive profile of the N-glycome in zebrafish endothelial cells. In order to explain the previously observed increase in HSPC numbers in microRNA *miR-223* mutants [117], the authors compared transcriptomic data on sorted miR-223 reporter-positive endothelial cells from wildtype and *miR-223* null embryos. They observed that several of the most significantly upregulated genes in the mutants, that also themselves contain miR-223 binding sites in the RNA transcript, were enzymes that regulate N-glycosylation of proteins [118]. Extraction and mass spectrometry of glycoproteins from sorted zebrafish endothelial cells showed that the N-glycan landscape was dysregulated, with a shift from high mannose- to high sialic acid-containing N-glycosylated proteins, and that the metalloprotease Adam10a was one such affected protein in zebrafish that regulates EHT. This study is an excellent example of layering proteomics on top of the transcriptomic and live-imaging data which are so readily obtained in the zebrafish model; future efforts in this space will likely push metabolomic and proteomic profiling of HE further to understand the cellular physiology underpinning the EHT process.

### 4.2. Hormones

**Figure 3:**
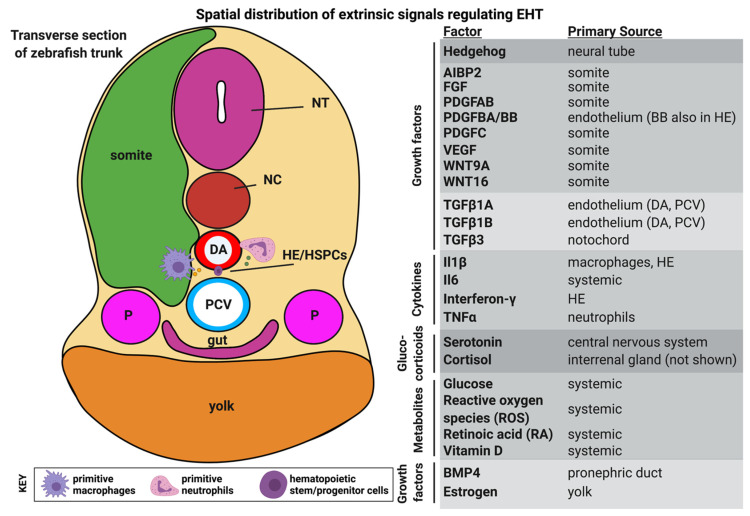
Spatial distribution of extrinsic signals regulating EHT. The cross-section on the left provides a whole-embryo context for the tissues surrounding the dorsal aorta from which HSCs will emerge from HE via an EHT process. To the right is a list of diffusible signaling molecules known to have functions in regulating HSC production. Some of these factors are present ubiquitously in the embryo or produced from multiple sources. DA dorsal aorta, HE hemogenic endothelium, HSPC hematopoietic stem/progenitor cell, NC notochord, NT neural tube, P pronephric duct, PCV posterior cardinal vein. Figure created with BioRender.com.

A number of diffusible small molecules have been shown to affect HSPC production from HE in zebrafish by stimulating nuclear hormone receptors and downstream signaling, Figure 3. Yolk-derived estrogen limits hematopoiesis by antagonizing Vegf signaling, helping demarcate the anatomical region of the aorta for HE specification [119]. Metabolites of vitamin D have differential effects on EHT. Unprocessed cholecalciferol (vitamin D3) restricts *runx1* and *cmyb* expression in the zebrafish by reducing the strength of the Hedgehog-Notch signaling axis independent of the vitamin D receptor (VDR) [120], while the biologically active metabolite 1,25(OH)D3 promotes HSPC production in zebrafish embryos and the function of human cord blood CD34^+^ cells in hematopoietic colony forming unit assays by the VDR-dependent upregulation of the chemokine Cxcl8 [121]. Thyroid hormone [122], cannabinoids [123] and glucocorticoids [124] have all been shown to modulate HSPC production in zebrafish models.

Another hormone-like compound with an emerging role in developmental hematopoiesis is the vitamin A metabolite, retinoic acid (RA). In brief, RA is a lipid-soluble signaling molecule that is synthesized in cells from vitamin A derivatives by retinaldehyde dehydrogenases (RALDH) and degraded by CYP26 enzymes [125]. RA binds and activates a class of nuclear hormone receptors that regulate transcription through RA-responsive DNA enhancers of target genes, including a number of HOX and CDX genes. In 2013, compelling evidence was provided for the tissue-specific necessity of RA synthesis by Raldh2 in the endothelium to produce definitive HSCs in mice [126]. Intriguingly, the original studies on RA signaling in zebrafish showed a limiting effect of exogenous RA specifically on the production of primitive blood-forming cells [127,128]. More recently, Pillay and colleagues concluded that RA signaling reduction in *aldh1a2*-deficient zebrafish (achieved by morpholino-knockdown or chemical inhibition of enzymatic activity) have reduced *cmyb* and thymic *rag1/ikaros* expression, consistent with compromised definitive blood stem cell production [129]. All of these studies relied on morpholino-based gene disruption, precluding temporal control of gene inactivation or dissection of contributions (by null allele analysis) from different RALDH enzymes or retinoic acid receptors. Future efforts should attempt to integrate these conflicting zebrafish data with recent advances related to the RA target HOX and CDX genes, and their potential to improve in vitro hematopoietic differentiation. The Sturgeon Lab has shown that CDX4 is required for the definitive potential of human pluripotent stem cell-derived HE in vitro [130]. Similarly, in fish, *cdx1a* and *cdx4* act redundantly to promote primitive blood cell development [131,132], at odds with the idea of them being RA targets in this context. The mechanisms delineating the “pro-definitive” and “anti-primitive” functions of RA exposure in zebrafish will likely require careful temporal- and tissue-specific pathway modulation with detailed analysis of cell fate and dynamic behaviors in live embryos to further clarify how this hormone instructs HSPC formation in vivo.

### 4.3. Sterile Inflammation

One of the most important roles of HSCs is ultimately the production and maintenance of the innate and adaptive immune system in vertebrates. Prolonged immune challenges and subsequent inflammatory responses in the adult can skew HSC differentiation in different hematopoietic lineages and affect the self-renewal capacity of the HSC pool. In the mid 2010′s, a windfall of publications provided zebrafish data establishing a role for non-pathogenic sterile inflammatory signaling promoting HSC production in the embryo. Implicated molecules included Tnfa/Tnfr2 [133], Interferon Gamma [134,135], and Tlr4/NfKB [136]. Ongoing efforts in this area have focused on identifying the full range of cytokines and endogenous processes that stimulate these inflammatory networks.

Recent progress has been made in linking glucose metabolism to the production of a number of inflammatory cytokines in promoting HSC development. Lim et al. demonstrated that glucose-associated Hif1a activity exerts its functions partially by inducing Il6 expression in HE downstream of Pdgfb [137]. A related study has delineated a mechanism for metabolism-directed inflammasome activation in the production and processing of the cytokine Il1b with positive effects on HSPC formation. By a combination of knockdown, knockout and chemical modulation of the NLRP3 inflammasome, Frame et al. [138] show that HSPC production in the zebrafish embryo is dependent on IL1B, with involvement of the inflammasome machinery in HE and macrophages to process this cytokine into its active form. Pharmacologic activation of the inflammasome with the compound nigericin from 24–120 hpf increased macrophage, neutrophil and T-lymphocyte populations in zebrafish embryos at the expense of a decreased erythrocyte pool. Notably, nigericin treatment was able to enhance multi-potent hematopoietic colony formation from in vitro human iPS-derived CD34^+^ cells in culture. An independent investigation similarly observed aberrant skewing of differentiation from HSPCs in zebrafish embryos exposed to inflammasome inhibition, namely a reduction in neutrophils and macrophages with a concomitant increase in erythropoiesis; however, these effects appeared to occur with no significant alterations in HSPC number [139]. This discrepancy may be attributable to differences in transgenic lines used for HSPC quantification and precise timepoints of analysis. Nevertheless, the NLRP3 inflammasome clearly can impact hematopoietic output from HE.

Exciting data from zebrafish have also emerged positioning nucleic acids as putative ligands to trigger sterile inflammatory regulation of hematopoiesis. Last year Lefkopoulos et al. [140] published that RIG-I-like receptors (RLRs) in HE cells are activated by transcribed RNAs from retroelements, inducing an inflammatory transcriptional signature via NfKB that buffers HSPC production from the aorta. RLRs function as RNA helicases and are known to respond to viral RNAs in pathogenic settings. The authors show that in zebrafish, knockdown of the RLRs *rig-l* and *mda5* reduces the number of HSPCs and the inflammatory gene signature in sorted HE cells. By analysis of retroelements expressed in HE gain-of-function experiments using *sine3-1a* RNA injections, they conclude that RNA from retroelements can positively regulate HSPC numbers via an RLR-dependent mechanism. A third RLR, Lgp2, serves to dampen HSPC production in the embryo via an unknown mechanism that does not involve inflammatory signaling. R-loops are yet another class of nucleic acid species shown this year to regulate hematopoiesis via inflammatory cues. R-loops are an entity consisting of a DNA:RNA hybrid and ssDNA, a byproduct of genomic transcription. The Bowman Lab demonstrated that the DEAD-box helicase Ddx41 limits HSPC production in zebrafish by clearing R-loops in the nucleus of HE cells, thereby preventing initiation of a cGAS/STING inflammation cascade [141]. *Ddx41* mutant zebrafish have enhanced HSPC numbers in the embryo, together with quantifiable increases in cellular R-loops. This phenotype could be restored by overexpression of RNASEH to reduce R-loops, or by knockdown of the cGAS/STING proteins, an inflammatory signaling axis upregulated in *ddx41* mutants. Collectively, these studies provide more clarity as to the physiological mechanisms underlying the sterile inflammatory regulation of hematopoiesis, potentially allowing these cues to be more finely tuned during in vitro hematopoietic differentiation to drive HSC production or expansion.

## 5. Extrinsic Cues: Finding New Relationships with ‘Omics Approaches

### 5.1. Bulk RNA-Sequencing: Hematopoietic Roles for GPCRs

Knowledge of the transcriptional profile of HE has been essential for identifying accurate markers of this population and in determining the molecular dysfunction at play during experimental conditions that perturb EHT (Table 1). RNA sequencing of immuno-phenotypic endothelial cells, HE, and HSCs in mice revealed a role for the cell surface receptor Gpr56 during EHT [142]. This gene was progressively expressed during the endothelial-HE-HSC trajectory, and knockdown of the homologous gene in zebrafish led to reduction in HSPCs in the embryo. These effects could be rescued by mRNA injection of either the zebrafish or mouse Gpr56 gene product. Maglitto et al. [143] have recently added to this story by showing a functional redundancy in the mouse for the Gpr56 and Gpr97 genes, bolstered by the ability of mouse Gpr97 to rescue Gpr56 knockdown in zebrafish.

A similar high-quality transcriptomic dataset of zebrafish endothelium, HE and HSPCs was generated by Zhang et al. [144] in 2015. The authors created a novel GFP reporter in zebrafish driven by a mouse Runx1-responsive hematopoietic enhancer. By combining this transgene with a pan-endothelial *Tg(flk1:mcherry)* line, a FACS enrichment strategy was used to isolate and sequence endothelial cells (GFP^−^, mCherry^+^), HE (GFP^+^, mCherry^+^) and HSPCs (GFP^+^, mCherry^−^) from the micro-dissected zebrafish trunk. In this study the *gpr183* gene was identified due to its relatively higher expression in the HE population, and Crispr/Cas9 mutagenesis demonstrated that zebrafish loss-of-function mutants had a reduction in cMyb-expressing cells around the timepoint of EHT and failure to acquire Rag1-expressing thymocytes at later timepoints. This group also showed conserved expression dynamics and function in mouse HE, in part by observing reduced hematopoietic output from explanted AGMs treated with a chemical inhibitor of the endogenous Gpr183 ligand.

Such datasets have been a powerful tool within the community to evaluate new experiments against and test hypotheses. Just last year, Kwon et al. [145] reported a hematopoietic role for yet another GPCR in zebrafish, Gpr182. They observed clear vascular expression of *gpr182* by whole mount in situ hybridization and determined from the Zhang dataset that transcript levels were particularly enriched in HE. RNA sequencing analysis of mutant endothelial cells and a chemical screen were used to identify LeukotrieneB4 as a putative ligand for this GPCR, and quantification of HSPC numbers in *gpr182* mutants showed increases over wildtype embryos, with a concomitant enhancement of myeloid differentiation. Together, these studies show how transcriptomic approaches have been employed to identify GPCRs in HE with both positive and negative effects on EHT and HSPC formation.

### 5.2. Tomo-Seq, scRNA-Seq and Ligand/Receptor Predictions

The previous studies identified cell intrinsic receptors (with extrinsic ligands) by bulk RNA sequencing of enriched HE cells. Limitations of this approach include an inability to parse cellular heterogeneity within the target population or identify genetically encoded extrinsic factors in the complex multicellular environment in which EHT occurs. Rapid progress has been made employing Tomo-seq and scRNA-seq technologies in zebrafish to gather regional and cell-type specific transcriptomic data and make computational predictions of cellular crosstalk in hematopoiesis.

One of the first applications of this approach in zebrafish was used to predict ligand/receptor crosstalk in the CHT niche. By laser microdissection and sequencing of regions of the CHT, Xue et al. [146] made predictions of ligand/receptor interactions by identifying endothelial-enriched receptors and cognate ligands on nearby tissue. Together with bulk and single-cell sequencing approaches, they uncovered Ctgfa/Itgb2 as a functional pair dampening HSPC expansion in this embryonic niche. In a cross-species effort aiming to specifically identify ligand/receptor interactions that regulate EHT, Yvernogeau and colleagues performed RNA tomography (Tomo-seq) on the AGM from stage-matched human, chick, mouse and zebrafish embryos [147]. They were able to then identify putative ligand/receptor pairs by conservation and endothelial expression between at least 2 or more species and could show in zebrafish novel hematopoietic functions for the Adm/Ramp2 ligand/receptor pair and the secreted growth factor Svep1. These studies provide exciting proof-of-concept for such computational predictions, and harbor as yet unvalidated ligand/receptor pairs that may regulate EHT.

### 5.3. Future Directions: Integrating Extrinsic Cues with Gene Regulatory Networks by Sequencing

scRNA-sequencing in particular has been used to identify rare populations of stem cells and infer differentiation trajectories and cellular states along the hematopoietic continuum from HE to HSCs and their progeny [148,149,150,151]. Transcriptomic profiling can therefore serve as a ‘quality control’ check, to determine how transcriptionally similar in vitro-derived or manipulated HSPCs might be to the native cell type; algorithms like CellNet [61], CellRouter [152], FateID [153] and SingleCellNet [154] are designed to provide such an analysis. High quality datasets from in vivo models are therefore essential. A number of groups have used scRNA-sequencing to profile zebrafish adult whole kidney marrow cells, providing a molecular atlas and gene signature for zebrafish HSCs and their derivatives [155,156,157]. This has been extended to HSPCs in the CHT as well [158].

Data for gene regulatory network (GRN) reconstruction are just beginning to be generated in human, mouse and zebrafish models. Bulk sequencing strategies for RNA transcripts and chromatin histone marks in mouse HE implicated hematopoietic roles for Sp3 and Maz TFs, with a conserved function shown for the zebrafish orthologs [159]. Bulk sequencing of zebrafish HE together with ATAC-seq by Bonkhofer et al. [160] provides strong molecular data reinforcing a transcriptional role for Runx1 in ultimately repressing the arterial fate in select HE cells to complete EHT. New algorithms are being developed to computationally predict ligand/receptor interactions occurring in tissues based on scRNA-seq data [161,162], but these tools have not been rigorously applied to hematopoietic development. Future efforts will likely focus on comprehensive integration of predicted extracellular cues (like ligand/receptor interactions or mechanical inputs) to GRNs in HE implied by the cell intrinsic TF milieu and chromatin landscape. Given the fruitfulness of cross-species comparison in the endeavor thus far, and the relative ease of functional validation, the need for high quality datasets in the zebrafish model is evident.

## 6. Conclusions

Endothelial cell heterogeneity is now understood to contribute to the functions of organ-specific vasculature and underlies the mechanisms by which the vascular tree is grown and shaped during development. Furthermore, much is known about the essential role of canonical developmental signaling and regulatory cascades (Notch, VEGF, WNT, TGF β/BMP, FGF, etc.) in the process of vascular specification and patterning, including that of hemogenic fate [165]. In this review, we have focused on the extrinsic signals derived from the hematopoietic niche microenvironment that further influence and modify cellular commitment to transition from vascular identity to blood formation. It is important to note, whereas this review has not covered the unique supportive functions of endothelial cells for HSC maintenance and function in adult and developmental niches, they remain a critical influence on the hematopoietic system. For example, bone marrow sinusoidal Apelin+ endothelial cells are required in mice for proper HSC number during steady-state and post-irradiation, transplantation-derived regenerative hematopoiesis [166]. In zebrafish, endothelial cells of the CHT have been observed to “cuddle” nascent HSPCs that are expanding in this transient embryonic stem cell niche [167], and the endothelium of this vascular bed expresses abundant growth factors to control retention, growth and development of stem cells [168]. In summary, we propose that hemogenic endothelium, and the developmental process of endothelial-to-hematopoietic transition, represents one of the most extreme specializations of an endothelial cell type, which when coaxed and presented with the right extrinsic and environmental cues will abandon the classical endothelial identity program completely to adopt a blood stem cell fate. Ongoing work in zebrafish and other systems stands to further decipher the molecular characteristics of the unique hemogenic endothelial cells and refine methods to recapitulate developmental processes to procure HSCs from this population, in vivo and in vitro.

## Figures and Tables

**Table 1:** RNA sequencing datasets of hemogenic endothelium and other hematopoietic tissues.

Last Author, Year	Species	Type of Sequencing	Sorted Population(s)	Accession Number(s)	Ref.
Zhang et al., 2015	zebrafish	bulk RNAseq	flk1:mCherry^+^ (ECs), flk1:mCherry^+^/ runx1en:GFP^+^ (HE); and runx1en:GFP^+^(HSPCs)	N/A	[144]
Kartalaei et al., 2015	mouse	bulk RNAseq	E10.5 AGM ECs(CD31^+^, cKit^−^,Ly6aGFP^−^), HE (CD31^+^, cKit^−^,Ly6aGFP^+^), HSCs(CD31^+^, cKit^+^,Ly6aGF^+^) and HPs( CD31^+^, cKit^+^,Ly6aGF^+^)	GSE63316	[142]
Bonkhofer et al., 2019	zebrafish	bulk RNAseq and ATACseq	*TgBAC(runx1P2:Citrine);Tg(kdrl: mCherry)* to sort for HE, non-HE arterial ECs and venous ECs, including *runx1* morphants	GSE132259, GSE132258	[160]
Baron et al., 2018	mouse	scRNA-seq	CD31^+^, cKit^+^ cells from E10 and E11 aorta after intra-aortic antibody staining, together with other aortic subfractions by surface markers	GSE112642	[148]
Zeng et al., 2019	human	scRNA-seq	Dissected AGM from ~30-day old human embryo (depleted of red blood cells)	GSE135202	[149]
Yvernogeau et al., 2020	zebrafish, mouse, chicken, human	Tomo-seq	zebrafish *Tg(kdrl:mCherry/cd41:EGFP)* at 28- and 40-hpf for HE/HSPC identification through trunk; E10.5 and E11.5 mouse trunk, E3 chicken embryo trunk, 35-day-old human embryo trunk	N/A	[147]
Chen et al., 2020	mouse	scRNA-seq	Lin^−^, cKit^+^ cells with both Runx1-mKO2 and Ly6a-GFP transgenic reporters (HSCs)	GSE145638	[150]
Zhu et al., 2020	mouse	scRNA-seq	Purified EC, HE and intra-aortic cluster cells with surface markers and Runx1-GFP expression	GSE137117	[163]
Oatley et al., 2020	mouse	scRNA-seq	VE-cadherin^+^ cells from E10 AGM	E-MTAB-6987	[164]
Kasper et al., 2020	zebrafish	scRNA-seq	Dissected trunks from 27hpf wildtype and miR-223 mutants, sorted on *Tg(kdrl:mCherry)* ECs (also has miR-223 GFP^+^ population for clustering)	GSE135246	[118]
Soto et al., 2021	zebrafish	scRNA-seq	*Tg(kdrl:EGFP)* ECs at 30hpf from *ezh1* wildtype, heterozygous and homozygous mutants	GSE173972	[56]

AGM: aorta-gonad-mesonephros, EC endothelial cell, HE hemogenic endothelium, HP hematopoietic progenitor, HSC hematopoietic stem cell, HSPC hematopoietic stem/progenitor cells.
